# Monitoring the Bystander Killing Effect of Human Multipotent Stem Cells for Treatment of Malignant Brain Tumors

**DOI:** 10.1155/2016/4095072

**Published:** 2016-01-06

**Authors:** Cindy Leten, Jesse Trekker, Tom Struys, Valerie D. Roobrouck, Tom Dresselaers, Greetje Vande Velde, Ivo Lambrichts, Catherine M. Verfaillie, Uwe Himmelreich

**Affiliations:** ^1^Biomedical MRI, Department of Imaging and Pathology, KU Leuven, 3000 Leuven, Belgium; ^2^Molecular Small Animal Imaging Center, KU Leuven, 3000 Leuven, Belgium; ^3^Imec, 3000 Leuven, Belgium; ^4^Morphology Research Group, Biomedical Research Institute, Hasselt University, 3590 Diepenbeek, Belgium; ^5^Department of Development and Regeneration, Stem Cell Institute, KU Leuven, 3000 Leuven, Belgium; ^6^Division of Radiology, University Hospitals Leuven, 3000 Leuven, Belgium

## Abstract

Tumor infiltrating stem cells have been suggested as a vehicle for the delivery of a suicide gene towards otherwise difficult to treat tumors like glioma. We have used herpes simplex virus thymidine kinase expressing human multipotent adult progenitor cells in two brain tumor models (hU87 and Hs683) in immune-compromised mice. In order to determine the best time point for the administration of the codrug ganciclovir, the stem cell distribution and viability were monitored* in vivo* using bioluminescence (BLI) and magnetic resonance imaging (MRI). Treatment was assessed by* in vivo* BLI and MRI of the tumors. We were able to show that suicide gene therapy using HSV-tk expressing stem cells can be followed* in vivo* by MRI and BLI. This has the advantage that (1) outliers can be detected earlier, (2) GCV treatment can be initiated based on stem cell distribution rather than on empirical time points, and (3) a more thorough follow-up can be provided prior to and after treatment of these animals. In contrast to rodent stem cell and tumor models, treatment success was limited in our model using human cell lines. This was most likely due to the lack of immune components in the immune-compromised rodents.

## 1. Introduction

Gliomas are the most common brain tumors in humans. They comprise a broad range of lesions with distinct differences in malignancy, which is assessed according to the World Health Organization classification [1]. Glioblastoma multiforme (GBM) is the most malignant glioma with a dismal prognosis despite the advantages in conventional therapy including complete surgical resection, chemotherapy, and radiotherapy [2, 3]. Tumor relapse is mainly due to infiltration of tumor cells into normal brain tissue and the presence of cancer stem cell populations [4–7]. In recent year, novel experimental treatment options have been considered and explored [3]. Gene therapy using viral vectors to transduce tumor cells with therapeutic genes is an attractive alternative to conventional therapy. Hereby, approaches range from mutation correction, enhancement of the immune response against tumor cells, RNA interference, and targeted lysis of tumor cells using selective replicative viruses, to antiangiogenic and suicide gene therapies [8, 9]. Several suicide genes have been tested successfully in experimental models. Hereby, the most extensively studied systems are the herpes simplex virus thymidine kinase gene (HSV-tk) with the prodrug ganciclovir (GCV) and the cytosine deaminase gene, which converts 5-fluorocytosine into the cytotoxic 5-fluorouracil [9, 10]. As infiltration of glioma cells into normal brain tissue makes delivery of the suicide gene difficult, new options have been explored to target these infiltrating cells [6, 11–13]. Hereby, neural and mesenchymal stem cells are a suitable vehicle for the suicide gene as these cells have the ability to migrate to malignant gliomas and track infiltrating tumor cells [5, 14–17].

This approach relies on the administration of cells carrying a suicide gene, such as HSV-tk. When a substrate like GCV is provided, it enters the cell and is converted by HSV-TK into GVC-monophosphate [6]. Subsequently, cellular kinases recognize the monophosphate and will create GCV-triphosphate, a guanine nucleoside analogue which causes DNA chain termination and subsequent cell death.

**Figure 1:**
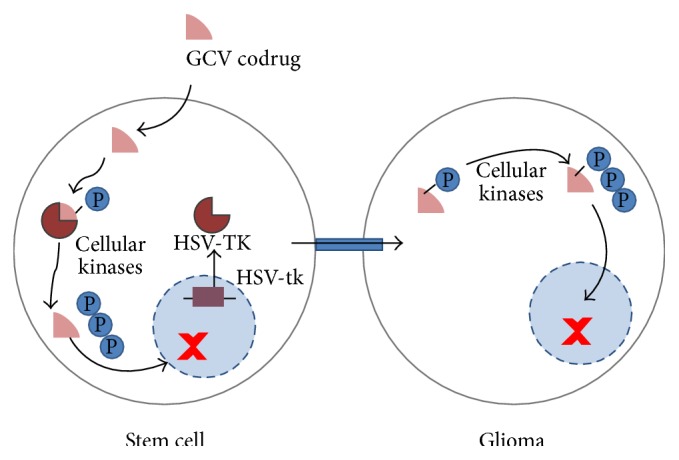
Concept of tumor therapy by using suicide gene expressing stem cells that are able to track tumor cells. It has been shown that certain stem cells are able to track infiltrating tumor cells [5, 14–17, 22]. In addition, the therapeutic cells must carry a suicide gene, in this case the herpes simplex virus thymidine kinase (HSV-TK). When a substrate for the HSV-TK enzyme, ganciclovir (GCV), is provided, it enters the cell and is converted by HSV-TK into GCV-monophosphate. The HSV-TK displays a 1000-fold higher affinity for GCV than the mammalian thymidine kinase so that systemic toxicity is limited while the increased affinity boosts tumor therapy capabilities [5]. Cellular kinases will phosphorylate the GCV-monophosphate further to GCV-triphosphate, a guanine nucleoside analogue which inhibits cellular DNA polymerase and results in chain termination with subsequent cell death. While this would erase the therapeutic cell but not the targeted tumor cell, a means for transferring the cytotoxic compound to the tumor cell is required. GCV-monophosphate can passively diffuse into neighboring cells after the formation of gap junctions between adjacent cells, which results mostly in tumor and therapeutic cell killing as normal adult brain cells usually do not replicate [36]. This is also known as “the bystander killing effect” [18, 37]. This approach can in theory terminate both primary and infiltrating tumor cells, thus eliminating sources of possible recurrent tumors [5].

Due to the formation of gap junctions between adjacent cells [18–20], GCV-monophosphate can passively diffuse into neighboring cells, which will mainly result in tumor and therapeutic cell killing as normal adult brain cells do not replicate. This is also called bystander killing effect (see also Figure 1) as tumor and therapeutic cells will be terminated. When using stem cells that can track infiltrating tumor cells, this method can in theory be applied not only to remove the main tumor but also to destroy any remaining tumor cells, thus eliminating sources of possible tumor recurrence [6]. Hereby, therapeutic cells are also eliminated after GCV administration, suppressing possible adverse effects like uncontrolled stem cell proliferation [21]. The feasibility of this strategy was demonstrated by several groups in both xenograft and syngeneic animal models [5, 21, 22].

Not only do noninvasive imaging strategies play an important role for the diagnosis and grading of brain tumors in humans [23–26], but they are also essential to follow up and assess the success of therapy* in vivo* [24]. This is important not only in the clinic but also for the assessment of experimental models. In contrast to diagnostic imaging procedures like magnetic resonance imaging (MRI) or positron emission tomography (PET), a wider range of imaging methods is available for preclinical research. In particular, optical imaging methods with limited depth penetration, like bioluminescence imaging (BLI) or fluorescent imaging, are extremely valuable in mouse tumor models as they are cost efficient and allow the monitoring of cell viability and gene expression [27–29]. In the field of (stem) cell research, it is also of advantage to monitor the location, viability, and gene expression of the therapeutic cells. Hereby, MRI, PET, and BLI are most frequently used [29, 30].

Previously, we have shown that suicide gene therapy using HSV-tk expressing mouse Oct4^−^ bone marrow derived multipotent adult progenitor cells (mOct4^−^ BM-MAPCs) as cellular vehicles can be validated by multimodal imaging using MRI and BLI [17]. Hereby,* in vivo* imaging methods were suitable not only for assessing therapy outcome but also for making therapeutic decisions. For example, if GCV is administered before stem cells have migrated towards the tumor cells, therapy may fail due to premature killing of the therapeutic cells. In the current study, it was our aim to validate this concept in experimental human glioma models (hU87 and Hs683) using therapeutic human multipotent progenitor cells (hMultistem) in immune-compromised animals. The distribution of stem cells labeled with superparamagnetic iron oxide particles (SPIO) was followed up by MRI. Their viability and suicide gene expression were assessed by using transgenic cells that express the firefly luciferase (fLuc) gene and the HSV-tk gene, respectively. Treatment was validated by monitoring the tumor volume (MRI) and tumor cell viability of Renilla Luciferase (rLuc) expressing hU87 or Hs683 glioma cells.

## 2. Materials and Methods

### 2.1. Cell Culture

The human bone marrow derived stem cell line hMultistem was obtained from ReGenesys BVBA (Heverlee, Belgium). The cells were cultured as described previously [31]. Cells were cultured at densities of 2 × 10^3^ cells/cm^2^.

The human brain tumor cell lines hU87 and Hs683 were obtained from the ATCC (Molsheim, France) and the laboratory of Dr. R. Kiss (Universite Libre de Bruxelles, Brussels, Belgium), respectively. The hU87 cell line was cultured using Eagle's Minimum Essential Medium (EMEM, Gibco Invitrogen) supplemented with 10% fetal bovine serum (FCS). Hs683 cells were cultured using RPMI 1640 (Gibco Invitrogen) supplemented with 10% FCS, 0.2% L-glutamine (Gibco Invitrogen), 100 units penicillin, 100 *μ*g streptomycin (Cellgro), and gentamycin (50 *μ*g/mL, Gibco Invitrogen).

### 2.2. Cell Transduction

Lentiviral vectors were provided by the Viral Vector Core, Molecular Virology and Gene Therapy, KU Leuven. hMultistem cells were consecutively transduced (p24 = 20 pg p24/cell) with two lentiviral vectors with an EF1*α* promoter [21]. First, a lentiviral vector encoding the eGFP gene and a puromycin resistance cassette were used to transduce the cells (LV-EF1*α*-eGFP-IRES-PuroR). Cells were split twice to allow the sufficient cell growth and transgene expression before selecting the cells using increasing concentrations of puromycin (2–8 *μ*g mL^−1^, Sigma-Aldrich, St. Louis, MO, USA). A second lentiviral vector encoding the fLuc, HSV-tk, and blasticidin resistance cassette genes was used to again transduce the cells (LV-EF1*α*-3flag-fLuc-T2A-HSV-TK-IRES-BsdR). Afterwards, cells were split twice before selection commenced using increasing blasticidin concentrations (20–80 *μ*g mL^−1^, Invivogen, San Diego, USA). Control cells were generated by adopting a similar strategy using the LV-EF1*α*-3flag-fLuc-T2A-eGFP-IRES-BsdR.

The human tumor cell lines hU87 and Hs683 were transduced using a CMV promoter driven lentiviral vector, which encodes for mCherry and rLuc. 1.25 × 10^4^/cm^2^ tumor cells were seeded and allowed to attach prior to transduction. Cells were incubated (108 pg p24/cell) with a CMV promoter driven lentiviral vector (provided by the Viral Vector Core, KU Leuven, Belgium) encoding for mCherry, rLuc, and a blasticidin resistance cassette for 24 hours. Subsequently, cells were washed and expanded. Approximately one week after transduction, mCherry expression became apparent. At that time, blasticidin selection commenced and was maintained for one week (40–60 *μ*g/mL). 2.5 × 10^4^ cells were seeded per well and allowed to attach after which rLuc BLI (IVIS 100 system, Perkin Elmer) was performed using coelenterazine-h (Rediject Coelenterazine-h, Perkin Elmer, 0.25 *μ*g/well) as a substrate.

### 2.3. *In Vitro* Confirmation of Transduction

For the hMultistem cells, eGFP expression was used for visual confirmation of transduction after which fLuc and HSV-tk expression were assessed. For fLuc expression, 100.000 cells were seeded in triplicate in a 24-well plate and were allowed to attach before BLI measurements were performed. An amount of 75 *μ*g of D-luciferin (Promega, Madison, WI, USA) was added per well prior to BLI experiments using an IVIS 100 system (Perkin Elmer, Waltham, MA, USA). The temperature was maintained at 37°C. Scanning parameters included medium binning,* f* stop = 1, time = 10 s or 1 min. Data were analyzed by using the living image 2.50.1 software. Similarly, mCherry expression was used for the tumor cell lines in order to confirm transduction by fluorescence microscopy. BLI was performed using the rLuc substrate coelenterazine-h (Rediject Coelenterazine-h, Perkin Elmer, 0.25 *μ*g/well).

HSV-tk expression was confirmed with a GCV killing experiment. Hereby, 20.000 cells were seeded in a 24-well plate and were allowed to grow. The following day, ganciclovir (Cymevene, Roche, Basel, Switzerland) was added in different concentrations (100 *μ*M, 1 *μ*M, and 0.01 *μ*M) for four consecutive days, after which BLI was performed. Cells were subsequently collected and a BCA protein assay (Thermo Scientific, Rockford, USA) was performed.

### 2.4. Cell Labeling with Superparamagnetic Iron Oxide Particles (SPIO) Labeling

hMultistem cells were labeled for 24 hours with in-house produced superparamagnetic iron oxide particles (SPIO) (seed-mediated growth; silane-functionalized and PEGylated (SMG^2^-mPEGSi) nanoparticles with a diameter of approximately 13 nm; 20 *μ*g iron/mL medium) in combination with poly-L-lysine (1.5 *μ*g/mL) [32, 33]. Subsequently, cells were washed three times and kept overnight in fresh medium after which 1 × 10^5^ cells were harvested for MRI and 5 × 10^4^ cells were harvested for ICP-OES measurements (for more details, see also [32, 33]). MRI agar phantoms containing 500 cells *μ*L^−1^ were prepared to validate MRI detectability limits.* In vitro *T_2_^*∗*^-weighted MR images were acquired (parameters: multigradient echo pulse sequence, repetition time (TR): 1500 ms, first echo time (TE): 4.44 ms with 8 increments of 6.75 ms, matrix size: 400 × 400, in plane resolution: 18.7 *μ*m^2^, slice thickness: 0.35 mm, and number of slices: 12).

### 2.5. Tumor Models: Stereotactic Cell Injection

All animal experiments were conducted according to the European Union Community Council guidelines and were approved by the local ethics committee of KU Leuven. Before surgery, animals were anaesthetized by an i.p. injection with a mixture of ketamine (4.5 mg/kg, Ceva, Pompidou, France)/medetomidine (0.6 mg/kg, Domitor, Pfizer, New York, USA). Local analgesia (2% xylocaine, AstraZeneca, London, UK) and antibiotics (6 mg/mouse, Ampiveto-20, 200 mg/ml, VMD, New Haw, Surrey, UK) were administered prior to surgery. After fixation of the animals in a stereotactic frame adapted with a quintessential stereotaxic injector (Stoelting, Wood Dale, USA), Hs683 or hU87 tumor cells were resuspended in 5 *μ*L PBS and injected (0.5 *μ*L min^−1^) into the right striatum of nude Hsd:Athymic-*Foxn1* ^nu^ mice using a 10 *μ*L Hamilton syringe, equipped with a 22 G needle. The following coordinates were chosen for cell injection: 0.5 mm anteriorly, 2.0 mm lateral to bregma, and 3.0 mm from the dura. Anesthesia was reversed using* Antisedan* (0.015 mg/animal, Pfizer Animal Health, Terre Haute, Indiana, USA). The number of animals is indicated in the respective figure legends.

### 2.6. Tumor Models (hU87 and Hs683): Suicide Gene Therapy

After injection of 3 × 10^5^ hU87 or 5 × 10^4^ Hs683 cells in the striatum of nude Hsd:Athymic-*Foxn1* ^nu^ mice, tumors grew for one (hU87) or two (Hs683) weeks prior to stereotactic intratumor injection of 5 × 10^5^ labeled and transduced hMultistem cells. Two weeks after tumor induction, three experimental groups were generated: (1) sham operated animals receiving no cells (PBS injection), (2) PBS treated control animals receiving 5 × 10^5^ hMultistems and treatment with PBS injections, and (3) GCV treated animals also receiving 5 × 10^5^ hMultistems but also treatment with GCV. Treatment (GCV (50 mg/kg) or PBS) was administered for 14 consecutive days starting at day 1 after hMultistem cell injection. At the end of PBS/GCV administration, both MRI and BLI were performed to assess tumor response and cell viability, respectively. Of the injected hMultistem cells, only 1% of cells were labeled with SPIO to reduce imaging artifacts [17]. Imaging was performed prior (MRI) to and following hMultistem injection (MRI/BLI). At the end of treatment, both MRI and BLI were performed to assess tumor response and hMultistem viability, respectively.

### 2.7. Imaging: MRI

All MR images were acquired using a 9.4 T BioSpec small animal MR scanner (Bruker Biospin, Ettlingen, GE) equipped with a horizontal bore magnet and an actively shielded gradient set of 600 mT m^−1^ (117 mm inner diameter) using a 7 cm linearly polarized resonator for transmission and an actively decoupled dedicated mouse surface coil for receiving (Rapid Biomedical, Rimpar, Germany). MRI was performed after tumor induction, prior to and following stem cell injection but before treatment initiation, and at the end of the treatment. Prior to scanning, mice were anaesthetized with 2% isoflurane for induction and 1.5% isoflurane for maintenance, respectively. Temperature and respiration were monitored throughout the experiment and maintained at 37°C and 100–120 breaths/minute. For* in vivo* tracking of the SPIO labeled hMultistem cells, 3D T_2_^*∗*^-weighted MR images (FLASH sequence, TR: 100 ms, TE: 12 ms, flip angle: 20°, resolution: 0.0078 cm/pixel, and field of view: 2.0 × 1.5 × 0.75 cm) were acquired and analyzed with the ImageJ software (National Institute of Health, Bethesda, Maryland, USA) to calculate the hypointense pixel volume. Furthermore, T_2_-weighted axial (RARE sequence, TR: 3157.6 ms, TE: 48.8 ms, matrix size: 256, FOV, 2.5 cm, in plane resolution: 0.0078 cm/pixel, number of slices: 24, and slice thickness: 0.05 cm) and coronal (RARE sequence, TR: 3000 ms, TE: 50.2 ms, matrix size: 256, FOV: 2.5 cm, number of slices: 15, and in plane resolution: 0.0078 cm/pixel) MRI scans were acquired to follow up tumor size. The area of the lesion was determined by outlining it manually on all slices using the 5.1 Paravision software (Bruker, Biospin). The sum of the cross-sectional area was used to determine the total tumor size. An ANOVA test (GraphPad PRISM, GraphPad Software, La Jolla, CA, USA) was used to determine significant differences with *p* < 0.05. To validate the integrity of the blood-brain barrier (BBB), pre- and postcontrast (Dotarem (100 *μ*L/mouse of 0.05 mmol/mL, i.v.), Guerbet, Villepinte, France) T_1_-weighted MR images were acquired (RARE sequence, TR: 819 ms, TE: 7.6 ms, matrix size: 256, FOV: 2 cm, number of slices: 20, and in plane resolution: 0.0078 cm/pixel). For statistical analysis of the blood-brain barrier integrity, the percentage of increase following Dotarem injection was calculated as previously described [34].

### 2.8. Imaging: BLI

Mice were anesthetized with isoflurane (2% induction, 1.5% maintenance) and placed in the IVIS 100 optical imaging system (Perkin Elmer), maintaining the body temperature at 37°C. Subsequently, D-luciferin (126 mg/kg, Promega) or coelenterazine-h (15 *μ*g/mouse, Perkin Elmer) were intravenously injected to assess stem cell or hU87 tumor cell viability, respectively. Subsequently, bioluminescent images were acquired. Data were analyzed for maximum intensity of the photon flux by the living image 2.50.1 software (Perkin Elmer).

### 2.9. Humane Endpoints

Animals were sacrificed when symptoms reached grade 3 out of 4 (grade 0 for healthy mice, grade 1 for slight unilateral paralysis, grade 2 for moderate unilateral paralysis and/or beginning hunchback, grade 3 for severe unilateral or bilateral paralysis and pronounced hunchback, and grade 4 for moribund mice) [35].

### 2.10. Histology

Animals were sacrificed by an i.p. overdose of Nembutal (300 *μ*L, Ceva) and subsequently perfused with 4% ice-cold paraformaldehyde (PFA) solution (Sigma-Aldrich). After overnight postfixation in 4% PFA, brain tissue was stored at 4°C in a PBS solution containing 0.1% sodium azide (Fluka, Sigma-Aldrich). Five *μ*m thick paraffin sections were cut and a Masson trichrome, a Prussian blue, and an Iba1 immunostaining were performed [32]. For the latter, sections were stained with an Iba1 antibody (1/250). Visualization was achieved by using the Dako EnVision + system-HRP (DAB) kit (Dako, Glostrup, Denmark).

### 2.11. Statistical Analysis

Statistical analysis was performed using GraphPad Prism (GraphPad Software PRISM, La Jolla, CA, USA). Significant differences between GCV and PBS treated animals regarding total tumor volumes (MRI: mm^3^) and cell viability (BLI: P/s) were determined by means of 2-way ANOVA test with differences of *p* < 0.05 regarded as significant. Figures show means ± SEM.

## 3. Results

The concept of the bystander killing effect using human bone marrow derived stem cells (hMultistems) that are capable of tracking brain tumor cells and that express a suicide gene is illustrated in Figure 1. A multimodal imaging approach was taken to monitor the fate of the stem and tumor cells to assess the success of therapy.

### 3.1. *In Vitro* Assessment of Transduction and Labeling of hMultistem and Tumor Cells

**Figure 2:**
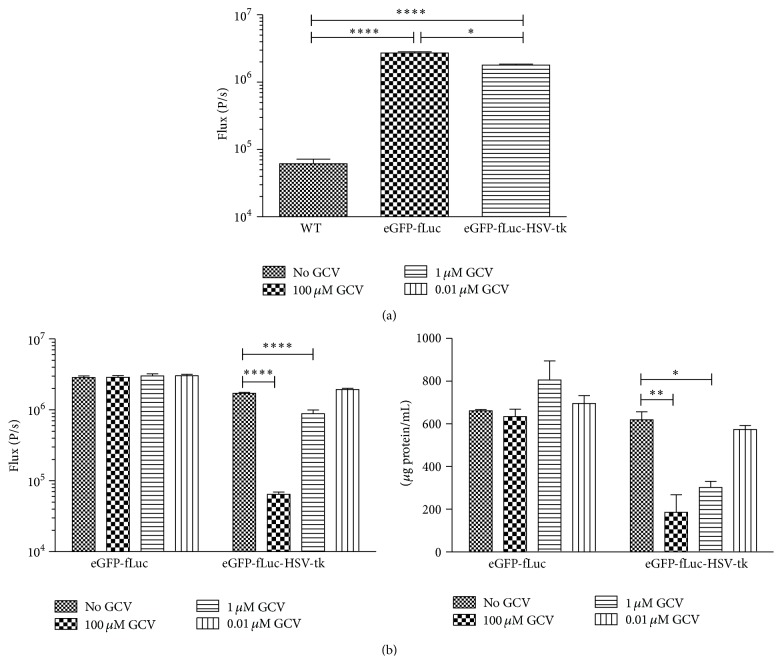
*In vitro* validation of hMultistem transduction. (a) Assessment of firefly luciferase (fLuc) expression via BLI showed a significant difference between transduced (eGFP-fLuc as well as eGFP-fLuc-HSV-tk) and wt hMultistem. (b) GCV killing experiment for validation of HSV-tk expression. Left: BLI signal was significantly decreased in eGFP-fLuc-HSV-tk expressing cells but not in eGFP-fLuc expressing control cells for all GCV concentrations compared to cells without GCV treatment. Right: the BCA protein assay confirmed BLI results and showed a dose-dependent cell killing in eGFP-fLuc-HSV-tk expressing cells but not in eGFP-fLuc expressing cells. Levels of significance are expressed as ^*∗*^ *p* < 0.05, ^*∗∗*^ *p* < 0.01, ^*∗∗∗*^ *p* < 0.001, and ^*∗∗∗∗*^ *p* < 0.0001.

The hMultistem cell line was transduced with a lentiviral vector in order to express either eGFP and fLuc (control) or eGFP, fLuc, and HSV-TK. The eGFP expression was used for visual confirmation of transduction after which fLuc and HSV-tk expression was assessed. BLI of cells transduced with either eGFP-fLuc (2.71 × 10^6^  ± 1.17 × 10^5^) or eGFP-fLuc-HSV-tk (1.79 × 10^6^  ± 5.83 × 10^4^) constructs showed a significant increased signal intensity compared to wild type (wt) hMultistem cells (6.16 ± 1.02 × 10^4^) (Figure 2(a)).

Subsequently, the HSV-TK expression was assessed by adding 0.01 *μ*M, 1 *μ*M, or 1 mM GCV to the cells during four consecutive days, which exerted a dose-dependent cell killing effect in eGFP-fLuc-HSV-tk expressing hMultistem cells but not in the eGFP-fLuc expressing control cells as assessed by BLI and a BSA protein assay.

**Figure 3:**
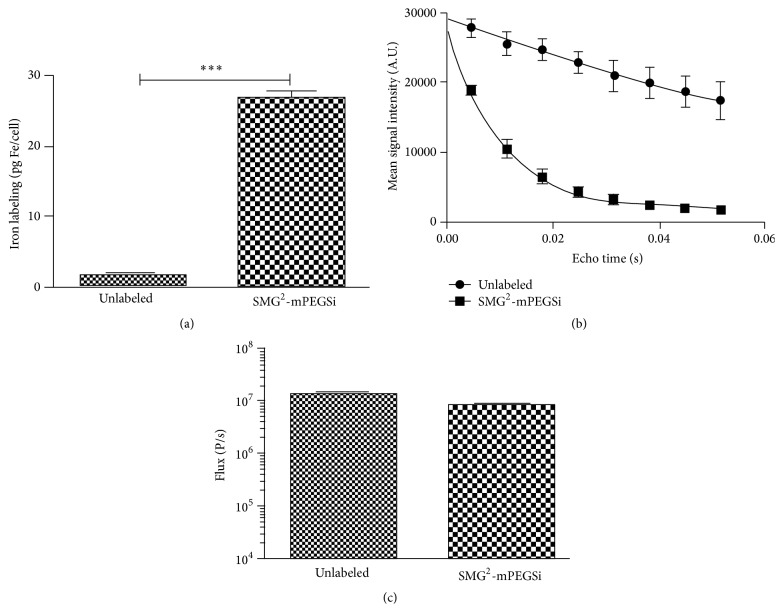
*In vitro* validation of SPIO labeling of hMultistem cells. (a) ICP-OES experiments confirmed iron internalization after SPIO labeling (10 ± 1 pg Fe cell^−1^) but not for unlabeled control cells. (b) Multiecho T_2_ map (MRI) confirms higher relaxation rats for SPIO labeled cells compared to controls. (c) BLI signal intensity was not significantly affected by SPIO labeling of cells (^*∗∗∗*^ *p* < 0.001).

In order to follow the distribution of engrafted hMultistem cells* in vivo* with high resolution, we decided to acquire 3D MR images. Therefore, eGFP-fLuc-HSV-tk^+^ hMultistem cells were labeled with in-house produced, biocompatible SPIO [32]. Detailed characterization of the labeled cells confirmed that uptake of SPIO in high quantity (TEM and ICP-OES), resulting in sensitive detection by MRI. The BLI signal intensity was not affected by the SPIO labeling (see Figure 3).

**Figure 4:**
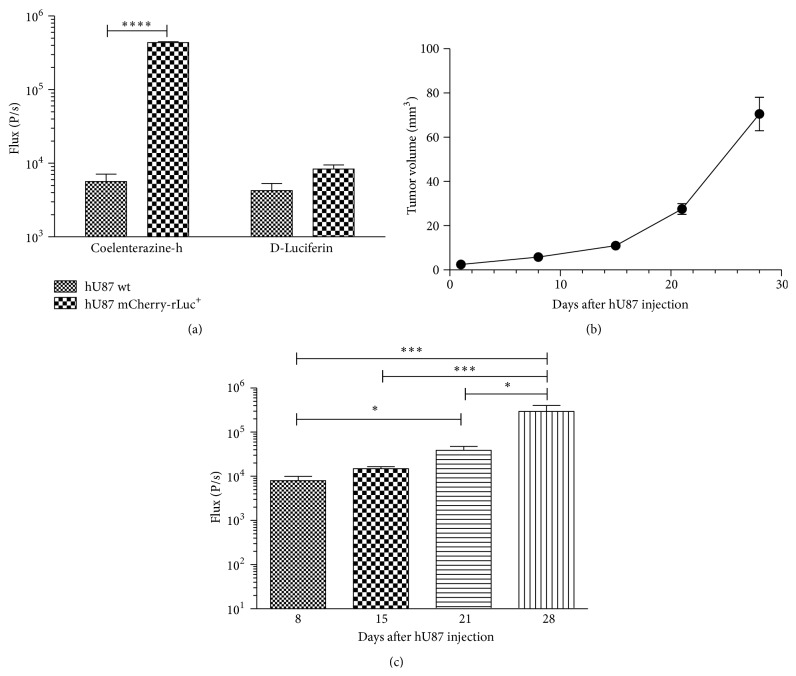
*In vitro and in vivo* validation of hU87 transduction with a mCherry-rLuc encoding vector. (a)* In vitro* analysis of mCherry-rLuc expressing hU87 cells showing sufficient expression of rLuc for* in vitro* detection by BLI using coelenterazine-h as a substrate. Furthermore, results show no statistically significant signal intensity differences after exposure of the rLuc positive hU87 cells to D-luciferin. (b) MRI-based tumor volume measurements from hU87 tumor bearing animals showed rapid growth with tumors reaching an average tumor size of 27.6 ± 2.4 mm^3^ at day 21 after injection. (c) BLI measurements on mCherry-rLuc positive hU87 tumor bearing mice also indicated tumor growth over time. Levels of significance are expressed as ^*∗*^ *p* < 0.05, ^*∗∗*^ *p* < 0.01, ^*∗∗∗*^ *p* < 0.001, and ^*∗∗∗∗*^ *p* < 0.0001.

In order to follow tumor growths and in parallel the fLuc expressing hMultistem cells in the hU87 model by BLI longitudinally, hU87 cells were transduced to express mCherry and rLuc.  Figure 4 indicates that the transgene expression can be assessed by BLI* in vitro*. These data prove that rLuc expression is sufficiently high for detection using coelenterazine-h but was not affected when using D-luciferin as a substrate. This implies that it is possible to distinguish the BLI signal originating from the hMultistem cells (fLuc-D-luciferin system) from the signal originating from the hU87 cells (rLuc-coelenterazine-h). To assess the transgene expression* in vivo*, 3 × 10^5^ transduced hU87 cells were injected in the striatum of nude Hsd:Athymic-*FoxN1* ^nu^ mice. Tumors grew rapidly with tumor size reaching 27.6 ± 2.4 mm^3^ by day 21 after injection (Figure 4(b)). Simultaneously, the BLI signal increased (Figure 4(c)), indicating tumor growth with detectable BLI signals from day 8 onwards (day one: 91.7 ± 90.7 P/s).

### 3.2. *In Vivo* Validation of hMultistem Survival and Suicide Gene Therapy

**Figure 5:**
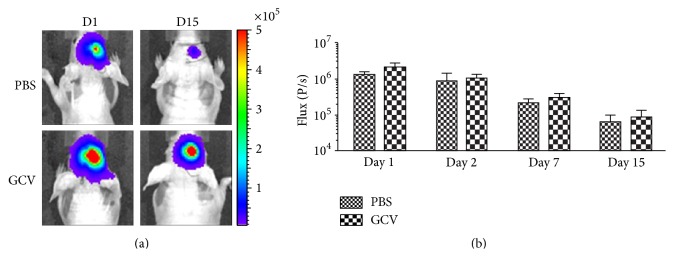
*In vivo* validation of BLI signal intensity and hMultistem survival in Hsd:Athymic-*FoxN1* ^nu^ mice. (a) Bioluminescence images of a representative animal that received 5 × 10^5^ hMultistems (day 1) and was treated from day 2 to day 15 with either PBS or GCV (50 mg/kg). (b) Quantification of BLI signal intensities shows that (1) the stem cells were detectable by BLI, (2) stem cell survival was reduced in the environment of the host's brain, and (3) no statistically significant difference in stem cell viability between the PBS and GCV receiving animals was detected. Number of animals was 6 per group.

In order to validate cell survival and GCV killing of hMultistem, 5 × 10^5^ transduced cells were stereotactically injected in the striatum of Hsd:Athymic-*FoxN1* ^nu^ mice. Subsequently, cell survival was assessed by BLI following PBS or GCV (50 mg/kg) injection for 15 days. As shown in Figure 5, cell viability decreased independently of PBS or GCV injection, indicating that stem cell survival is compromised in the host environment. One possible explanation for only marginal differences of BLI signal intensities between PBS and GCV treated animals is the lack of GCV to cross an apparently intact blood-brain barrier (BBB).

### 3.3. Validation of hMultistem Mediated Suicide Gene Therapy in a Humanized Oligodendroglioma Model (Hs683)

**Figure 6:**
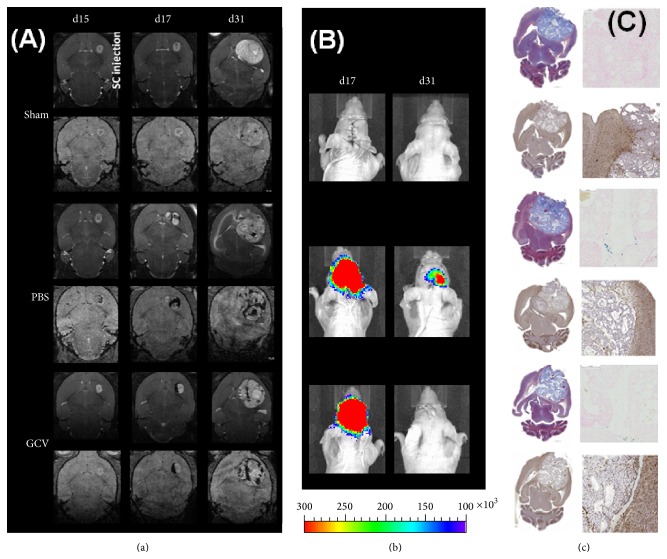
Follow-up of suicide gene therapy using hMultistem as cellular vehicles in the Hs683 oligodendroglioma model by using multimodal* in vivo* imaging and histology. (a) MR images of a representative animal from the sham, PBS, and GCV treated groups show a comparable tumor growth prior to hMultistem injection on T_2_-weighted MR images (upper row for each group). While there is little hypointense contrast visible on 3D T_2_^*∗*^-weighted MR images (lower row for each group), the distribution of SPIO labeled hMultistem cells could clearly be detected due to their hypointense contrast in the PBS and GCV treated group. When tumors grew larger, the hypointense voxels got more dispersed over time. (b) BLI of representative animals. The fLuc expression of the stem cells was clearly detectable after engraftment and only diminished after 14 days. (c) One week after the end of therapy, animals developed symptoms after which histological analysis was performed. Masson's trichrome staining (upper left) of all animals showed very large tumors. Prussian blue (upper right) staining was also performed which confirmed the presence of iron in PBS and GCV treated animals. Finally, Iba1 staining (bottom) was performed which showed a predominant absence of activated microglia in all treatment groups. Some minor microglial activation was however detectable around the tumor.

In order to validate the crossing of GCV through the BBB in tumor models, 5 × 10^5^ transduced and SPIO labeled hMultistem cells were stereotactically injected in the striatum of Hs683 tumor bearing nude Hsd:Athymic-*FoxN1* ^nu^ mice followed by daily administration of PBS (control) or GCV (50 mg/kg) for 14 consecutive days. Histological, BLI, and MR images of representative animals are shown in Figure 6.

**Figure 7:**
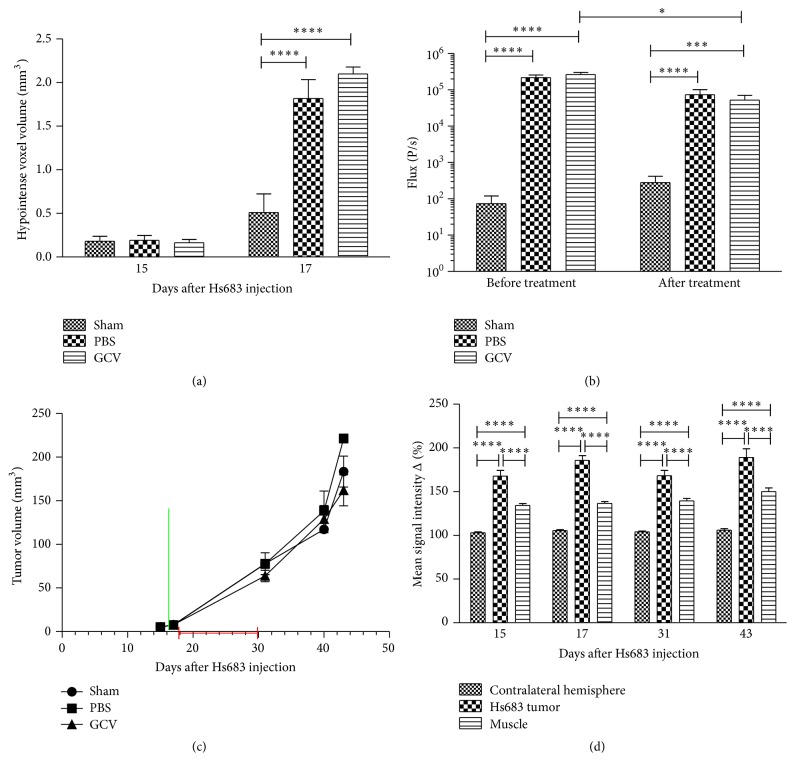
Quantification of* in vivo* imaging data to monitor suicide gene therapy using hMultistem as cellular vehicles in the Hs683 oligodendroglioma model. (a) Quantification of the volume of hypointense voxels in animals receiving SPIO labeled hMultistem cells indicates sufficient contrast to detect the therapeutic cells in the Hs683 tumor model (sham (no cells): *N* = 3, PBS treated: *N* = 6, and GCV treated: *N* = 6). (b) Bioluminescent imaging data showed that the stem cells were detectable through BLI, but no statistically significant difference in stem cell viability between the PBS and GCV receiving animals could be detected following treatment. However, there was a small decrease in the BLI signal before and after treatment in the GCV treated group. (c) Tumor volume measurements proved no statistical differences between sham operated, PBS treated, or GCV treated animals (green bar: hMultistem injection; red bar: duration of GCV/PBS treatment). (d) T_1_-weighted MRI pre- and postcontrast injection showed BBB integrity loss in the Hs683 tumor compared to the contralateral hemisphere and the extracranial muscle at all time points (*N* = 15). Levels of significance are expressed as ^*∗*^ *p* < 0.05, ^*∗∗*^ *p* < 0.01, ^*∗∗∗*^ *p* < 0.001, and ^*∗∗∗∗*^ *p* < 0.0001.

MRI data confirmed correct administration and distribution of hMultistem cells, which surrounded the tumor lesion following stereotactic injection (Figures 6(a) and 7(a)). BLI data confirmed that the expression of the fLuc gene was sufficiently high for* in vivo* detection of the hMultistem cells. However, only a small decrease in BLI signal intensity was observed after GCV, but not PBS treatment (Figure 7). No statistically significant difference in cell viability (BLI signal intensity, Figure 7(b)) could be observed between GCV treated and PBS treated animals after treatment.

Concurrent with these results, no statistically significant differences in tumor volume were found between the sham operated (*N* = 3), PBS treated (*N* = 6), and GCV treated (*N* = 6) animals at the end of the treatment (day 31), nor in the subsequent progression of tumor growth (Figure 7(c)). Developing tumors showed statistically significant signal enhancement in T_1_-weighted MR images after administration of contrast agent, confirming disruption of the blood-brain barrier (Figure 7(d)). Histological analysis confirmed the* in vivo* imaging results (Figure 6(c)). Prussian blue staining showed iron accumulation only in PBS and GCV treated animals, most likely arising from the SPIO labeled stem cells. Staining with Iba I for the presence of activated microglia was performed, which showed remarkably few activated microglial cells inside the tumor when compared to previous results using mouse stem cells in a mouse tumor model [17].

### 3.4. Validation of hMultistem Mediated Suicide Gene Therapy in a Humanized Glioblastoma Model (hU87)

One possible reason for the partial failure of bystander mediated suicide therapy in the Hs683 model is the fact that this model shows only a limited disruption of the BBB (for Gd-based chelates but not for Evans blue staining) [34]. Therefore, we have also validated the suicide gene therapy approach using hMultistem cells in the generally accepted hU87 glioblastoma model of astrocytic origin.

After establishment of the tumors, 5 × 10^5^ eGFP-fLuc-HSV-tk expressing SPIO labeled hMultistem cells were stereotactically injected in the striatum of mCherry-rLuc expressing hU87 tumor bearing nude Hsd:Athymic-*FoxN1* ^nu^ mice. Thereafter, PBS or GCV (50 mg/kg) was administered for 14 consecutive days.

**Figure 8:**
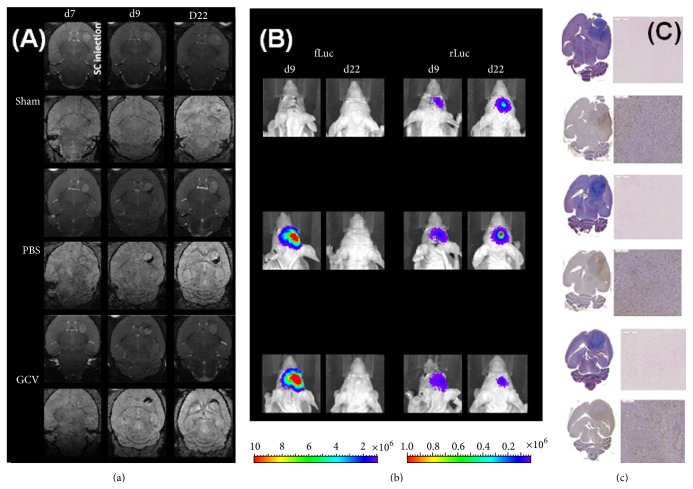
Follow-up of suicide gene therapy using hMultistem as cellular vehicles in the hU87 glioma model by using multimodal* in vivo* imaging and histology. (a) MR images of a representative animal from the sham, PBS, and GCV treated groups show a comparable tumor growth prior to hMultistem injection on T_2_-weighted MR images (upper row for each group). While there is little hypointense contrast visible on 3D T_2_^*∗*^-weighted MR images (lower row for each group), the distribution of SPIO labeled hMultistem cells could clearly be detected due to their hypointense contrast in the PBS and GCV treated group (one day after engraftment). The contrast distribution changed only very little until the end of treatment. When tumors grew larger, the hypointense voxels got more dispersed over time. (b) Left: bioluminescent imaging using D-luciferin as a substrate for fLuc expressing hMultistem cells. Stem cells were detectable after engraftment but not after the end of treatment for both, the PBS and GCV receiving groups. Right: bioluminescence imaging using coelenterazine-h as a substrate for rLuc expressing hU87 tumor cells. The signal intensity increased for the sham and PBS group but not for the GCV group, indicating some inhibition of tumor growth. (c) Two weeks after the end of therapy, animals developed symptoms after which histological analysis was performed. Masson's trichrome staining (upper left) of all animals showed large, dense tumors. Prussian blue (upper right) staining was also performed which confirmed the presence of iron in PBS and GCV treated animals. Finally, Iba1 staining (bottom) was performed which showed a predominant absence of activated microglia in all treatment groups.

**Figure 9:**
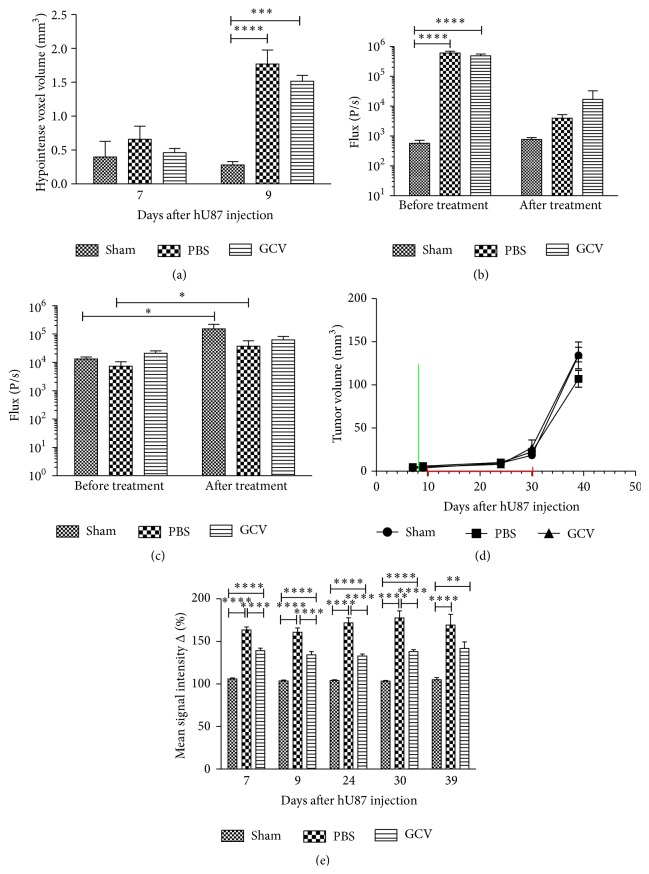
Quantification of* in vivo* imaging data to monitor suicide gene therapy using hMultistem as cellular vehicles in the hU87 glioblastoma model. (a) Quantification of the volume of hypointense voxels in animals receiving SPIO labeled eGFP-fLuc-HSV-tk^+^ hMultistem cells indicates sufficient contrast to detect the therapeutic cells in the hU87 tumor model (sham (no cells): *N* = 3, PBS treated: *N* = 5, and GCV treated: *N* = 6). (b) Bioluminescent imaging data using D-luciferin as a substrate for fLuc expressing hMultistem cells showed that the stem cells were detectable through BLI. A decrease in stem cell viability was detected for both PBS and GCV treated animals, which indicates that the stem cells are unable to survive well in the tumor microenvironment. (c) Bioluminescent imaging data using coelenterazine-h as a substrate for rLuc to investigate tumor viability indicated tumor growth in the sham operated and PBS treated group but not in the GCV treated group. (d) Tumor volume measurements proved no statistical differences between sham operated, PBS treated, or GCV treated animals (green bar: hMultistem injection; red bar: duration of GCV/PBS treatment). (e) T_1_-weighted MRI pre- and postcontrast injection showed BBB integrity loss in the hU87 tumor compared to the contralateral hemisphere and the extracranial muscle at all time points (*N* = 11). Levels of significance are expressed as ^*∗*^ *p* < 0.05, ^*∗∗*^ *p* < 0.01, ^*∗∗∗*^ *p* < 0.001, and ^*∗∗∗∗*^ *p* < 0.0001.

MRI data confirmed that the distribution (MRI) and viability (BLI) of hMultistem cells could be determined* in vivo* following stereotactic injection. Representative images are shown in Figure 8. Quantitative data are shown in Figure 9. Unexpectedly, a decrease of the fLuc BLI signal intensity of the engrafted hMultistem was seen not only for GCV treated animals but also for the PBS treated animals, indicating that the tumor environment may have induced cell death of the injected hMultistem cells. The BLI signal intensity of the rLuc expressing hU87 tumor cells showed an increase of the BLI signal for the sham operated animals and the PBS treated group but not for the GCV treated group when comparing signal intensities before and after initiation of the treatment (Figure 9(c)), indicating decreased tumor cell viability. However, the inhibition of tumor growth compared to the two control groups was relatively small as also confirmed by the MRI-based determination of tumor volumes, where no statistically significant differences between the sham operated, PBS treated, and GCV treated groups were found (Figure 9(d)). This decreased viability might however reflect a slight increase in the size of necrotic areas, which is difficult to assess on MRI due to T_2_^*∗*^ effects caused by bleeding and the SPIO labeled hMultistem cells. Tumors showed statistically significant enhanced signal intensity in T_1_-weighted MRI when comparing pre- and postcontrast conditions (Figure 9(e)).

## 4. Discussion

Although numerous efforts have been made to ameliorate the prognosis for glioblastoma patients, results have been limited so far [38]. During the past decades, suicide gene therapy has been investigated as a new therapeutic approach. Unfortunately, results obtained in clinical trials have been limited, mainly due to insufficient expression and poor intratumoral distribution of the viral vectors encoding for the suicide gene [39]. Therefore, HSV-tk expressing stem cells that are able to track infiltrating tumor cells are a promising vehicle for suicide gene delivery to the target site [5, 6, 11–13]. Hereby,* in vivo* imaging methods are used not only to assess the success of therapy but also to make therapeutic decisions. For example, if the codrug GCV is delivered before the therapeutic (stem) cells have reached the targeted tumor cells, treatment might fail due to premature termination of the therapeutic vehicle. Assessment of the location and viability of the stem cells prior to initiation of treatment is possible by using MRI, PET, or BLI [28–30]. In addition, tumor size and tumor cell viability can also be assessed by using clinical (MRI, PET, and SPECT) or experimental (BLI, photoacoustic imaging, and fluorescence imaging) techniques [24, 25]. For stem cell based therapy approaches, knowledge of the exact location of the tumor is also important to guide cell engraftment. In this way, animals can be followed longitudinally, thus reducing the number of animals needed. Additional information can be generated to gain a more thorough understanding of the dynamics of this experimental glioblastoma treatment. As multiple parameters have to be acquired almost simultaneously, multimodal imaging approaches are of advantage providing additional, clinically relevant parameters and allowing parallel monitoring of different cell populations [25, 28, 29].

In this study, Hs683 and hU87 tumor bearing nude Hsd:Athymic-*FoxN1* ^nu^ mice were stereotactically injected with 5 × 10^5^ HSV-tk/FLuc expressing and SPIO labeled hMultistem cells for longitudinal follow-up of suicide gene therapy. Three groups were studied, which included a sham operated group (no stem cells), a PBS treated group (stem cells but no codrug), and a GCV treated group (50 mg kg^−1^ daily for 2 weeks). Based on MRI and BLI, the treatment commenced after distribution of the therapeutic cells around the tumor. Stem cell viability was verified by BLI before and after treatment. The distribution of the stem cells around the tumor was confirmed by histology. The hMultistem cell viability decreased in both the PBS and the GCV treated group in control animal (no tumor) and the hU87 tumor bearing animals but only to a smaller extent in the Hs683 tumor bearing animals as assessed by BLI using D-luciferin as a substrate for fLuc, indicating that some of the loss of viable stem cells can be attributed to the hostile microenvironment in the host. No differences could be found in either tumor model concerning tumor size between animals that were sham operated or treated with PBS or GCV. However, there was a stagnation of hU87 tumor growth after GCV treatment, which was not the case in PBS treated or sham operated animals. This therapeutic effect was detected by BLI but not by anatomical imaging (MRI), which typically show a delayed response to treatment effects.

When compared to animal models that used mouse or rat tumor and stem cells [5, 17], stem cell mediated suicide gene therapy in humanized tumor models proved much less effective. There are several plausible explanations for this failure of therapeutic response. First, developing tumors at the time of stem cell injection were generally larger in the hU87 and Hs683 models compared to the GL261 mouse model, which may require the injection of more stem cells to elicit a therapeutic response. However, at least a delay in tumor growths should have been detected, which was only the case in the hU87 model. Secondly, the bystander killing effect depends on the formation of gap junctions between adjacent cells. It has been reported that different brain tumor cells display variable gap junction formation, which might result in variable, cell line specific therapeutic response [20, 40]. Thirdly, the success of HSV-tk based gene therapy also depends on the successful delivery of the codrug GCV to the target site. After engraftment of stem cells in brains without tumors, no difference between PBS and GCV treated animals was seen, most likely due to the failure of GCV crossing the BBB. Although disruption of the BBB was demonstrated in our tumor models using Gd-based contrast agents, the extent of the BBB disruption may still vary as demonstrated before [34]. Finally, it is believed that the bystander killing effect is also in part T cell mediated [41–43] which would partially explain the absence of therapeutic effects in the T cell lacking humanized mouse models. This theory is also supported by our observation that in some cases PBS treated GL261 tumor bearing animals, which received m-Oct4(−)-BM-MAPCs, also show a decreased tumor growth even when clear tumor growth was present at the time of stem cell injection [17].

Our results indicate that although stem cell based suicide gene therapy is a promising approach, further investigations are required to better understand possible mechanisms of action in different tumor environments using a variety of stem cells and host environments to optimize the therapeutic effect. In order to better understand the role of the immune system in our model system, it would be advisable to further study the successful m-Oct4(−)-BM-MAPCs/GL261 system in mice with a compromised immune system. With the development of the mentioned imaging techniques, it is now possible to monitor dynamic changes in different model systems longitudinally* in vivo*, providing a rapid screening approach to optimize stem cell therapy in malignant glioma models.

In conclusion, we have shown that suicide gene therapy using hMultistem as cellular carriers can be monitored* in vivo* by MRI and BLI. Hereby, MRI can be used to ascertain stem cell location prior to treatment initiation and tumor growth follow-up. BLI can be used to assess stem cell and tumor cell viability prior to and following treatment. Thus, outliers can be detected earlier, GCV treatment can be initiated based on stem cell distribution rather than on empirical time points, and a more thorough follow-up is possible prior to and following treatment of these animals. This will hopefully lead to a better understanding and optimization of a promising therapeutic approach for glioblastoma patients.
